# Etiology, Clinical Manifestations, Diagnosis, and Treatment of Cobalamin (Vitamin B12) Deficiency

**DOI:** 10.7759/cureus.52153

**Published:** 2024-01-12

**Authors:** Sakshi S Jajoo, Udit M Zamwar, Prachee Nagrale

**Affiliations:** 1 Endocrinology, Jawaharlal Nehru Medical College, Datta Meghe Institute of Higher Education and Research, Wardha, IND; 2 Ophthalmology, Jawaharlal Nehru Medical College, Datta Meghe Institute of Higher Education and Research, Wardha, IND

**Keywords:** transcobalamin, macrocytic anaemia, intrinsic factor, pernicious anemias, cobalamin deficiency

## Abstract

Cobalamin, also known as vitamin B12, is a water-soluble vitamin. Cobalamin deficiency can be frequently seen in people all around the world. It can have non-specific symptoms, and in patients who are in a very critical state, it can lead to neurological or hematological abnormalities. While pernicious anemia used to be the main cause, it now accounts for a smaller number of cases, with food-bound cobalamin malabsorption being more common. Early diagnosis and appropriate management are crucial to avoid severe complications like spinal cord degeneration and pancytopenia. The primary method of treatment has been injections of vitamin B12 which are given through the intramuscular route but now the oral replacement therapy has also been very effective in treating the patients. There is increasing evidence linking increased levels of vitamin B12 to hematological and hepatic disorders, particularly cancers. This review has primarily highlighted the metabolism, clinical manifestations, diagnosis, and treatment of cobalamin deficiency in the past decade.

## Introduction and background

Vitamin deficiencies are a widespread issue globally, and deficiency of cobalamin has been recognized as a health concern for almost a century. Vegetarians are more prone to cobalamin deficiency than non-vegetarians. The main presenting symptom of cobalamin deficiency is peripheral neuropathy. Deficiency of cobalamin has been defined based on the test used for its diagnosis [1]. Diagnosis is based on a test for levels of vitamin B12 that are below 148 pmol/L along with clinical features of deficient cobalamin, or vitamin B12 levels below 148 pmol/L along with increased homocysteine or methylmalonic acid (MMA) levels [2]. In the United States, the occurrence of cobalamin deficiency varies by age group, affecting around 3%-6% of individuals in different age ranges. Marginal depletion, with cobalamin levels of 148-221 pmol/L, affects approximately 15%-20% of individuals in certain age groups.

The occurrence of cobalamin deficiency is different in various age groups in the United States, affecting at least 3% of people aged 20 to 39 years, 4% of people aged 40 to 59 years, and 6% of people aged 60 years and older. In this study, marginal depletion was defined as a serum vitamin B12 level between 148 and 221 pmol/L, and it affects 15% of people between the ages of 20 and 59 years and more than 20% of people above 60 years [3].

## Review

Methodology

**Figure 1:**
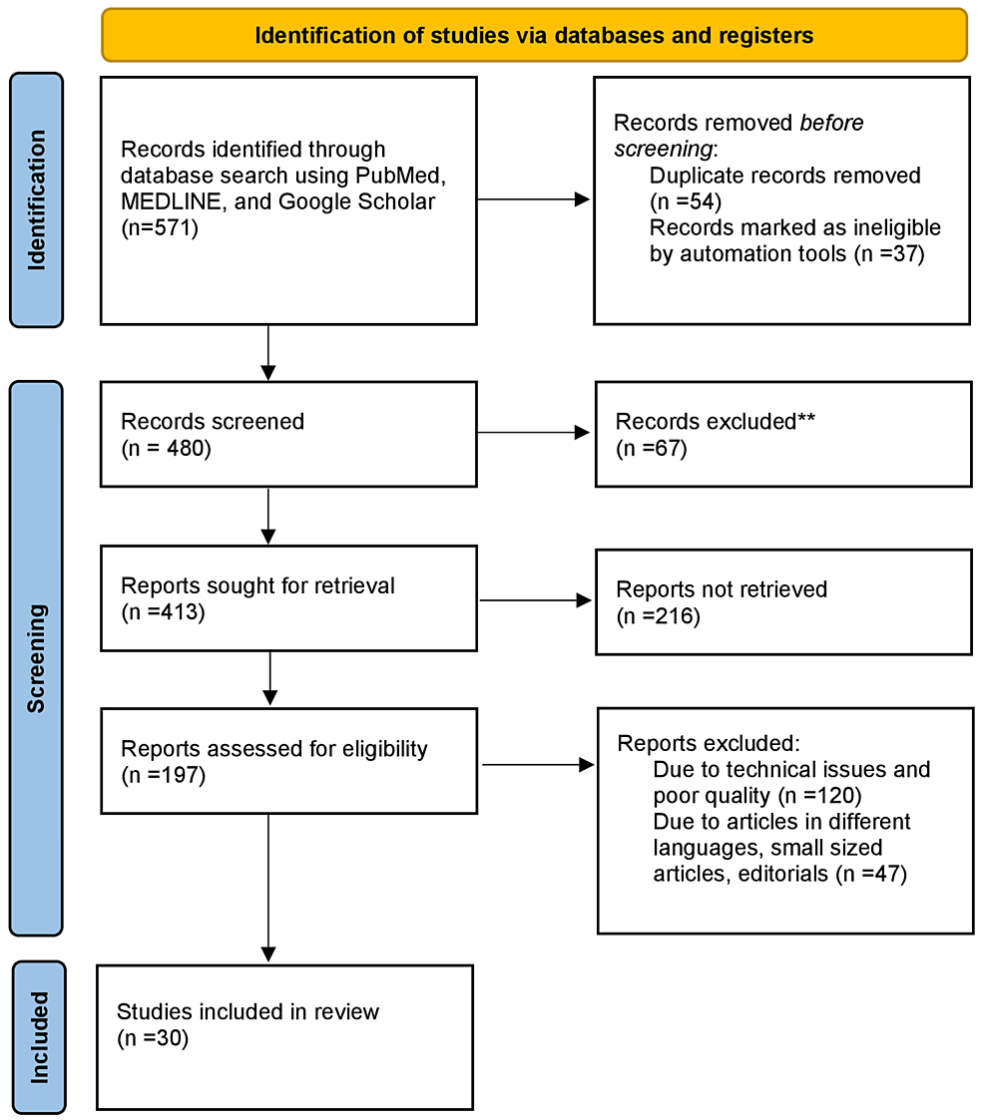
PRISMA methodology used in the study PRISMA: Preferred Reporting Items for Systematic Reviews and Meta-Analyses

Published articles were searched on the databases of MEDLINE, PubMed, and Google Scholar. The query terms were “vitamin B12” OR “cobalamin”; “etiology” OR “cause”; “treatment” OR “modalities”. This article includes the researches conducted on vitamin B12 deficiency, the etiology and clinical features of vitamin B12 deficiency, and different treatment methods. Research conducted since the previous 10 years have been depicted in this review article. The Preferred Reporting Items for Systematic Reviews and Meta-Analyses (PRISMA) method used in research methodology is depicted in Figure 1.

Cobalamin metabolism

Fish, milk and milk products, and red meat are considered to be rich sources of naturally gained cobalamin [4]. People have an average intake of 2.3 mcg of vitamin B12 per day from food sources, but only 50% to 60% is absorbed by the body in total [5]. To enhance absorption in the small intestine, dietary cobalamin is found in close proximity to proteins present in the food. It should be released upon coming in contact with the acidic pH of the stomach. When vitamin B12 is released, haptocorrin (transcobalamin I) binds it right away and keeps it there until the complex is broken up by proteolytic enzymes like proteases in the duodenum. The second carrier protein, intrinsic factor (IF), which is produced by the oxyntic cells of the gastric mucosa, can be found here. The terminal ileum's ability to absorb vitamin B12 depends on IF. Vitamin B12 separates from IF as it crosses the brush boundary and takes entry into the bloodstream, where it binds to transcobalamin I or II. Vitamin B12 is delivered to peripheral tissues and the liver by haptocorrin and transcobalamin II [6].

Intracellular Metabolism

After being delivered to the tissues on the periphery, free vitamin B12 is produced. When homocysteine and methyltetrahydrofolate (N5-MeTHF) are combined in the cytosol, the methionine synthase (MS) uses cobalamin as a cofactor to create methionine and tetrahydrofolate (THF). The clinical signs of shortage are explained by the downstream creation of purines and pyrimidines provided by THF synthesis, which are necessary synthesis of RNA and DNA. The only other vitamin B12-dependent process is methylmalonyl CoA mutase, which converts methylmalonyl CoA into succinyl CoA in the mitochondria [7].

Large quantities of cobalamin are retained in the liver after absorption, as a result, any decline in the intake of cobalamin may take five to ten years to show clinically. One to five percent of the free cobalamin is passively absorbed from the intestine via a process called passive diffusion that is not dependent on the IF. This encourages the use of oral medications for the treatment of vitamin insufficiency as opposed to intramuscular injections [8].

Etiology

There are numerous ways that a vitamin B12 deficit might arise due to the complicated methods of absorption. The autoimmune death of oxyntic cells and subsequent reduction of IF secretion are the causes of the classic form of “pernicious anemia” [9]. Pernicious anemia used to account for a high number of cases of cobalamin insufficiency, despite being a well-known cause [10]. Different causes of vitamin B12 deficiency are depicted in Table 1 [1,2].

**Table 1:** Causes of cobalamin (vitamin B12) deficiency

Cause	Examples
Low intake of cobalamin	This cause is seen in people with a vegetarian diet, chronic alcoholics, and elderly people.
Autoimmune disorders	This cause is seen in people with Sjogren’s syndrome and pernicious anemia.
Food-bound cobalamin malabsorption (FBCM)	This cause is seen in people with atrophic or chronic gastritis, and Helicobacter pylori-associated gastritis.
Genetic disorders	This cause is seen in people who are deficient for transcobalamin II.
Cobalamin malabsorption	This cause is seen in people with celiac disease, achlorhydria, and Crohn’s disease.
Gynecological factors	This cause is seen in pregnant females and due to the use of oral contraceptives.
Surgical procedures	This cause is seen in people who have undergone partial or complete gastrectomy, and resection of the ileum.
Drugs	This cause is seen in people who take certain drugs like metformin and proton pump inhibitors.

Nowadays, FBCM has become a prevalent factor causing vitamin B12 deficiency [1]. The absorption of cobalamin from meals is compromised in FBCM. The inability to release cobalamin from its transport proteins is one of the factors that is present in many of the situations that might cause FBCM. A few conditions that diminish the production of hydrochloric acid (HCl) include stomach inflammation (gastritis), no secretion of HCl (achlorhydria), partial or total gastrectomy, and the use of proton pump inhibitors like pantoprazole or antacids like calcium carbonate, etc. These conditions reduce the amount of cobalamin that may be released from dietary proteins [8]. The milder symptoms seen in these patients can be attributed to an IF-dependent transport mechanism that is still functioning in this situation. According to a recent study, using proton pump inhibitors or histamine H2-receptor antagonists increases the risk of developing cobalamin deficiency in the future [11]. This risk is dose and time-dependent. The danger considerably lowered after the medicine was stopped. Thus, widespread use of acid-suppressing medications might result in cobalamin insufficiency, which might go undetected due to ignorance. According to de Jagar, who discovered that metformin use is linked to a considerable drop in vitamin B12 levels and a higher risk of future deficiency, metformin use also has a comparable effect on the levels of cobalamin [12]. Uncertainty surrounds the relationship between cobalamin insufficiency and the clinical symptoms. A recent comprehensive study emphasized the link between metformin use and a decrease in serum vitamin B12 levels [13].

Clinical manifestations

Hematological and neurological effects of cobalamin deficiency range from lesser symptoms like weariness and tingling or pricking sensations to severe ones like a decrease in levels of red blood cells (RBCs), white blood cells (WBCs), and platelets also known as pancytopenia, and spinal cord atrophy [10,14]. The clinical presentation of cobalamin deficiency is depicted in Table 2 [10,14].

**Table 2:** Clinical manifestations of cobalamin deficiency

Type	Clinical Presentation
Hematological	The patient presents with decreased haemoglobin (Hb) levels [10], hyper-segmented neutrophils [10], pancytopenia [10], decreased quantity of neutrophils and platelets in blood [10], and increased mean corpuscular volume (MCV) [10].
Neurological	The patient presents with peripheral neuropathy [14], erectile dysfunction [14], and spinal cord degeneration [14].
Neuropsychiatric	The patient presents with depression [14], delirium [14], mania [14], and Alzheimer’s disease [14].

There is no clear link between hematological and neurological characteristics, hence people with neurological symptoms may not also have a hematopoietic disorder and vice versa [15]. Given that neurological symptoms are less frequently used to diagnose vitamin B12 deficiency than macrocytic anemia, a sizable portion of patients who are suffering from cobalamin deficiency may go undiagnosed and run the risk of suffering from irreversible neurological complications. Patients suffering from cobalamin deficiency may be more susceptible to osteoporosis, according to some data [16]. Gastric cancer and other autoimmune diseases such as Hashimoto’s disease, diabetes mellitus type I, and vitiligo are all possible side effects of pernicious anemia [10]. The link between high blood homocysteine levels and atherosclerosis is well known, while the one between decreased vitamin B12 levels and atheromatous disease is still up for debate. There is proof that a cobalamin shortage does not raise the risk of heart disease, despite the concurrent rise in homocysteine levels reported with deficient cobalamin levels [17].

Diagnosis

The presence of a hypersegmented type of neutrophils in macrocytic anemia is indicative of cobalamin deficiency. The standard confirmatory test is the measurement of blood vitamin B12 levels. This test has a high sensitivity for the diagnosis if the results are under 148 pmol/L [2]. Given the overlap in metabolic pathways, it is advised that vitamin B12 and vitamin B9 (folate) levels be measured together [2].

Falsely low levels of cobalamin can be seen in multiple myeloma, haptocorrin deficiency, folate deficit, and oral contraceptive medication [18]. In renal insufficiency, serum MMA concentrations may be higher, which can complicate interpretation [14]. Due to the lack of any “gold standard” diagnostic test for cobalamin insufficiency, the clinical condition of the patient and the findings from the reports must both be taken into account.

Tests for Pernicious Anemia

Testing for serum autoantibodies is required to distinguish pernicious anemia from the other causative factors of decreased vitamin B12 levels [5]. In contrast to the anti-PC antibody test, which is highly sensitive (more than 90%) but has a specificity of just 50%, the anti-IF antibody test is comparatively not much sensitive (50%-70%) but has high specificity (>95%) [8]. Due to this, the British Committee for Standards in Hematology (BCSH) recommends against assessing anti-PC antibody titers and instead recommends the anti-IF antibody assay [2]. The Schilling test was used to rule out IF-mediated cobalamin malabsorption, although it is not presently available for clinical use [5]. Sadly, there is no diagnostic currently available for FBCM.

Some new tests have been introduced recently. The majority of tests for determining the amounts of serum cobalamin measure total vitamin B12, or vitamin B12 bound to both transcobalamin I and II [19]. In more recent testing, the amount of transcobalamin II coupled to cobalamin can be measured [6]. This type of test can be used to detect malabsorption of cobalamin after orally consuming it to measure its uptake by the intestine.

Treatment

People suffering from cobalamin deficiency in the United Kingdom are at present being given treatment with intramuscular injections of vitamin B12 by the guidelines provided, regardless of the underlying etiology [20,21]. The common treatment given to patients who are not suffering from any neurological damage includes the administration of 1 mg of hydroxocobalamin on alternate days for 14 days, followed by administration of three injections per month of the medicine [21]. This dosage schedule has to be followed for rest of the patient’s life if the patient is suffering from pernicious anemia. If there is another underlying cause for the deficiency, treatment should go on till any positive changes are seen in the blood profile of the patient. In case of patient suffering from any neurological problems, the dosage is continued until no further relief is seen clinically, after which two injections are given to the patient per month. If there are serious neurological symptoms, the patient is pregnant, or if there is any doubt about the test results or diagnostic procedure, referral to secondary care is advised [21]. If there is a possibility of celiac disease, carcinoma of the stomach, or malabsorption, gastroenterology tests are necessary. Managing underlying disorders, such as giving antibiotics for bacterial overgrowth, and stopping harmful medications can frequently be helpful [5].

Oral Cobalamin Medications

Despite the “reliance” on intramuscular delivery, mounting data suggests that oral vitamin B12 therapy may be just as successful. When the alternative IF independent mechanism for cobalamin absorption was discovered 60 years ago, it was found that large doses of 100-100,000 g were enough to meet the daily requirement, even though only 1% of the cyanocobalamin was absorbed [22]. In two different trials, Andrès et al. more recently showed the effectiveness of oral vitamin B12 in improving both the biochemical and clinical signs of cobalamin deficiency [23,24]. Following the daily delivery of 2,000 g of oral cyanocobalamin, a sustained increase in serum cobalamin levels was noticed. At a four-month follow-up, the serum levels were even higher as compared to those that produced in response to parenteral therapy [22]. Additionally, a comparable percentage of participants in both groups showed improved results in neurological symptoms such as bilateral paresthesia and impaired coordination [25-27]. Using the administration of 1,000 g orally each day, Castelli et al. produced comparable results in a trial using a similar strategy [25,28]. The convenience for the patient, lower medical expenses associated with delivering injections, and lower chances of bleeding in individuals who may be anti-coagulated are all clear benefits of oral therapy [26,29]. Even though oral cobalamin is not advised for treating pernicious anemia, it may be used to maintain or rectify an asymptomatic vitamin B12 deficit, according to the most recent BCSH guidelines [2,30]. The BCSH also suggests that FBCM patients may be given a low-dose (50 g) oral cobalamin prescription.

Subclinical cobalamin deficiency

Apart from the symptomatic insufficiency of vitamin B12, there is another condition known as “subclinical cobalamin deficiency” (SCCD) when there are biochemical abnormalities but no clinical signs [20]. Compared to clinical cobalamin deficiency, SCCD is more prevalent. In 30%-40% of cases, SCCD results from FBCM rather than being linked to an IF-mediated incapacity to absorb cobalamin. Currently, the consensus is that SCCD is a temporary condition that does not result in an overt cobalamin shortage. Nevertheless, a search must be started to rule out curable reasons. Patients who have serum cobalamin levels between 110 and 148 pmol/L should have their levels examined again in one to two months and those whose levels return to normal at that time do not need any additional testing [2]. Low-dose oral cobalamin should be administered as a backup to individuals whose cobalamin levels are consistently below acceptable levels, along with an anti-IF antibody titer test. It is advised that these people see their physician again if any neurological problems appear. The patient should be treated for pernicious anemia if anti-IF levels are positive. If they are negative, cobalamin levels should be re-evaluated after three to four months, and if they are consistently low, additional biochemical testing is necessary to confirm a biochemical deficiency [20]. Findings from the various studies are included in Table 3.

**Table 3:** Findings of studies included in this review article

Author Name	Year	Findings
Devalia V, et al. [2]	2014	Provides guidelines for the efficient diagnosis and treatment of vitamin B12 and folate deficiency in the people and establishes the correlation between cobalamin and folate deficiency.
Allen LH [3]	2009	Discusses the prevalence rate of cobalamin deficiency in the people and difference in the rates in different age groups.
Antony AC [4]	2003	Discusses the levels of cobalamin in people with different types of diets and focusses on cobalamin deficiency in people with vegetarian diet.
Nielsen MJ, et al. [6]	2012	Demonstrates the mechanism of absorption and transport of cobalamin gained from the food to the cells of the body and concludes that this process occurs in multiple steps.
Andrès E, et al. [8]	2004	Discusses the cobalamin deficiency found in older people, the reasons behind it and concludes that cobalamin deficiency is very common in older people.
Lam JR, et al. [11]	2013	Discusses the importance of proton pump inhibitor and its use in cobalamin deficiency.
Jager J, et al. [12]	2010	Discusses the prolonged use of the medication ‘metformin’ in patients suffering from type 2 diabetes mellitus and its effects on the cobalamin levels of the body.
Oijen MG, et al. [17]	2007	Explores the relation between hyperhomocysteinaemia and cobalamin deficiency, and the effects of prolonged deficiency on the cardiovascular system.
Chatthanawaree W [18]	2011	Discusses the various biomarkers involved in cobalamin deficiency and the use of these biomarkers for knowing the status of cobalamin deficiency in the human body.
Carmel R [20]	2012	Discusses the mild variant of cobalamin deficiency known as ‘subclinical cobalamin deficiency’ and its prevalence in the humans.
Kuzminski AM [22]	1998	Discusses the use of oral therapy with cobalamin to treat vitamin B12 deficient patients and its benefits on the patient health.
Castelli MC, et al. [25]	2011	Compares the oral and intramuscular treatment methods of cobalamin deficiency, and the merits and demerits of both.
Ebbing M, et al. [30]	2009	Discusses the incidence rate of carcinoma after treatment by administering cobalamin in patients and mortality rate of these patients after this treatment method.

## Conclusions

Deficiency of cobalamin is very frequently found in people all around the globe. People become more susceptible to cobalamin deficiency as their age increases. Etiology suggests that the most common cause of cobalamin deficiency is FBCM. FBCM only causes mild deficiency. Pernicious anemia causes severe deficiency but is very rarely found in people nowadays. Cobalamin deficiency is more common in vegetarians than non-vegetarians. The main symptoms of cobalamin deficiency include peripheral neuropathy, delirium, etc. The deficiency of cobalamin along with folic acid is the main cause of megaloblastic anemia. The diagnostic tests for cobalamin deficiency cannot be fully relied upon as there is no “gold standard” test present for its diagnosis. Symptoms of megaloblastic anemia strongly indicate cobalamin deficiency. Taking a good and complete history and looking for clinical manifestations is very necessary. There is a need for biomarkers like holotranscobalamin in the future, for finding the cause of cobalamin deficiency and providing proper and more accurate treatment. Intramuscular injections of cobalamin are the main method of treatment. Oral tablets of cobalamin are an emerging treatment method for cobalamin deficiency. Oral tablets provide a safer and more comfortable mode of treatment for patients instead of injections and hopefully someday may replace the injections.
